# Genetic Characterization of Sandfly-Borne Viruses in Phlebotomine Sandflies in Iran

**DOI:** 10.3390/microorganisms11112754

**Published:** 2023-11-11

**Authors:** Nariman Shahhosseini, Sarah-Jo Paquette, Mohammad Hassan Kayedi, Mohammad Reza Abaei, Mohammad Mehdi Sedaghat

**Affiliations:** 1 Department of Biological Sciences, University of Lethbridge, Lethbridge, AB T1K 3M4, Canada; sarahjo.paquette@alumni.uleth.ca; 2 Razi Herbal Medicines Research Center, Department of Parasitology and Mycology, School of Medicine, Lorestan University of Medical Sciences, Khorramabad 6814993165, Iran; kayedi78@yahoo.co.uk; 3 Department of Medical Entomology and Vector Control, School of Public Health, Tehran University of Medical Sciences, Tehran 141556446, Iran; abaimr@tums.ac.ir

**Keywords:** phlebovirus, sandfly, Karimabad virus, phylogenetics, Iran

## Abstract

Phleboviruses are classified into two main groups: the sandfly fever group (transmitted by sandflies and mosquitoes) and the Uukuniemi group (transmitted by ticks). Old World sandfly-borne viruses (SBVs) are classified into four main serocomplexes; sandfly fever Naples viruses (SFNVs), sandfly fever Sicilian viruses (SFSVs), Karimabad viruses (KARVs), and Salehabad viruses (SALVs). This study addresses current knowledge gaps on SBVs in Iran by focusing on identification and molecular epidemiology. We used PCR to examine DNA/RNA extracts to identify sandfly species and evaluate for SBV presence. We identified five specimens positive for phleboviruses: one *Ph. sergenti* for Tehran virus (TEHV), one *Ph. papatasi* for SFSV, and two *Ph. papatasi* and one *Ph. perfiliewi* for KARV. A phylogenetic tree indicated that the TEHV isolate from this study formed a cluster with previous isolates of TEHV, Zerdali virus, and Fermo virus. Meanwhile, the identified SFSV isolate fell in lineage I and was grouped with previous isolates of SFSVs and Dashli virus in Iran. Finally, the KARV isolates from this study formed a monophyletic clade in a sister relationship with other viruses in KARV lineages I and II. This comprehensive study on SBVs in Iran provided new insights into the molecular epidemiology of TEHV, SFSVs and KARVs in this country.

## 1. Introduction

Iran is a country rich in biodiversity, including various vectors known to transmit human diseases, such as sandflies [1]. Through the act of feeding on wildlife, phlebotomine sandflies can disseminate various pathogens, which include sandfly-borne viruses (SBV) [1,2,3]. Several SBVs are phleboviruses, which have a trisegmented, negative-sense, and single-stranded RNA genome [2,3]. The RNA-dependent RNA polymerase is encoded by the L gene, the viral envelope glycoproteins (designated Gn and Gc) are encoded by the M gene, and both the viral nucleocapsid protein (N) and nonstructural protein (NSs) are encoded by the S gene [4].

Viruses in the genus *Phlebovirus*, family *Phenuiviridae*, are classified into two groups based on their vectors: the sandfly fever group (transmitted by sandflies and mosquitoes) and the Uukuniemi group (transmitted by ticks) [5]. Within the Sandfly fever group, there are several SBVs of public health importance as well as the Rift Valley fever virus (RVFV), a mosquito-borne virus that has veterinary importance [6]. The Uukuniemi group is known for several human diseases, specifically severe fever with thrombocytopenia syndrome virus, Heartland virus, and Bhanja virus.

The Old World SBVs are mainly transmitted by *Phlebotomus* sandflies, while the New World SBVs are transmitted by *Lutzomyia* sandflies [7]. Old World SBVs can cause a range of diseases from neuroinvasive infections to self-limiting febrile diseases (also called sandfly fever) [4]. SBVs are extensively dispersed over the Mediterranean Basin, Africa, the Indian subcontinent, the Middle East, and the former USSR [8]. Entomological studies in Iran revealed the presence of 44 different sandfly species belonging to the genera of *Phlebotomus* and *Sergentomyia* [9].

SBVs that are grouped as “Old World” are classified into four main clades/serocomplexes [6], including sandfly fever Naples viruses (SFNVs), sandfly fever Sicilian viruses (SFSVs), Karimabad viruses (KARVs), and Salehabad viruses (SALVs) [10]. All four different SBV serocomplexes have been identified in Iran through virus isolation [11,12]. SFSV was isolated from an unidentified *Phlebotomus* spp. in 1975, and it was assumed that the unidentified sandflies might be *Ph. papatasi* due to the high prevalence (99%) of this species in collected samples. SALV was isolated from an unidentified *Phlebotomus* spp. in 1959, and KARV was first isolated from an unidentified pool of *Phlebotomus* spp. in 1959. In 1975, following KARV isolation from an unidentified pool of *Phlebotomus* spp. where 99% of samples were *Ph. papatasi,* it was presumed that *Ph. papatasi* might be the vector for KARV. Tehran virus (TEHV; belonging to the SFNV serocomplex) was isolated from *Ph. perfiliewi* in 1976 [11,12].

After initial SBV isolation in Iran, the presence of neutralizing antibodies in human sera collected from seven provinces of Iran demonstrates that SFSV (9.4–21.8%), SFNV (13.2–30.4%), and KARV (0.2–62.1%) were highly prevalent throughout the country in the 1970s [11,13]. Although the pathogenicity of KARV is unknown, specific antibodies were found in humans and other vertebrates in Pakistan [14], Bangladesh [15], and former Soviet Union countries [16]. In contrast, SALV-neutralizing antibodies were not detected in humans [4,11]. Since then, most studies on sandflies in Iran have focused on their role as a vector for the circulation of leishmaniasis, and not on their role as a vector for SBVs. Therefore, to fill the current knowledge gap on SBVs in Iran, the main objective of this study was to screen field-collected sandflies for phleboviruses and to subsequently identify the species of sandfly that carried the SBVs using molecular techniques.

## 2. Materials and Methods

### 2.1. Study Area and Sample Collection Methodology

**Figure 1:**
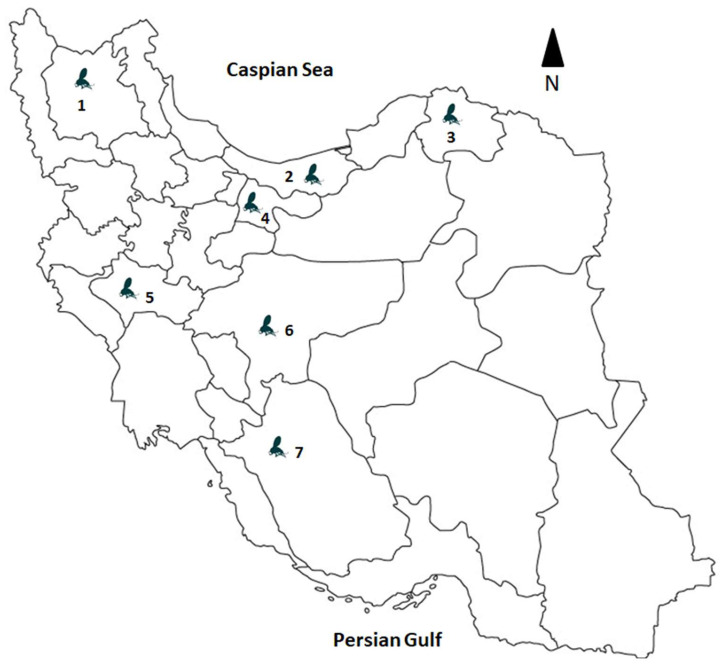
Trapping sites for sandflies collection during the period 2019–2020 in Iran. Sampling locations include site 1: East Azerbaijan Province (Osku), site 2: Mazandaran Province (Babol), site 3: North Khorasan Province (Esfarayen), site 4: Tehran Province (Abardej), site 5: Lorestan Province (Pole Dokhtar), site 6: Isfahan Province (Najafabad), and site 7: Fars Province (Kazerun).

Sandfly specimens were collected from May through September 2019 and 2020 at seven trapping locations across the seven provinces in Iran, including East Azerbaijan (site 1: Osku), Mazandaran (site 2: Babol), North Khorasan (site 3: Esfarayen), Tehran (site 4: Abardej), Lorestan (site 5: Pole Dokhtar), Isfahan (site 6: Najafabad), and Fars (site 7: Kazerun) (Figure 1).

Various traps (BG Sentinel traps, CDC light traps, and sticky traps) were set up in the vicinity of rodent tunnels, livestock dwellings, and residential areas to collect sandflies, as well as other insects for parallel research projects [17]. Sandfly samples were collected twice a week by changing the collection nets on all BG Sentinel and CDC light traps. The traps were in operation for the whole trapping period (24 h/day for 5 months). Sticky traps were replaced twice every week. After collection, specimens were delivered to the vector biology research Laboratory, Tehran University of Medical Sciences, in a cool box with ice packs and kept in a freezer at −20 °C until further analysis [18].

### 2.2. Homogenization and Gene Extraction

Specimens were homogenized individually by adding 1.0 mm zirconia beads (10 beads/sample) (Hunan, China) and 350 μL of phosphate-buffered saline (PBS). Samples were homogenized using a Tissuelyser (Qiagen, Hilden, Germany) for 3 min at 50 oscillations/s and subsequently centrifuged. After centrifugation, 17 μL supernatant from 10 individual specimens was used to generate pools. After initial pooling, 15 μL of solution from 10 pools was combined to generate super pools, each representing 100 individual specimens. RNA was extracted from 140 μL of super pools using the QIAamp Viral RNA kit (Qiagen, Hilden, Germany), based on the manufacturer’s protocol. Subsequently, DNA was extracted from 180 μL of individually homogenized samples and found to be positive for viruses using the DNeasy Blood & Tissue Mini Kit (Qiagen, Germany), as previously conducted [19].

### 2.3. Sandfly Molecular Identification of Virus-Positive Pools

Sandfly specimens were identified using molecular methods since morphological identification requires sample mounting, which can hinder downstream molecular procedures and analysis. Molecular identification of individual specimens within positive pools employed the amplification of a 499 bp segment of the mitochondrial cytochrome b (cytb) gene, as described in a previous study [20]. Amplicons were sequenced, and BLAST (http://blast.ncbi.nlm.nih.gov/Blast.cgi, accessed: 4 July 2021) was used to identify the sandfly specimens at the species level.

### 2.4. Pan-Phlebovirus and Specific RT-PCR

Samples were initially screened for phleboviruses with a Pan-RT-PCR using Superscript III (Invitrogen, Thermo Scientific, Bremen, Germany) and primers forward (TTTGCTTATCAAGGATTTGATGC), F2 (TTTGCTTATCAAGGATTTGACC) and reverse (TCAATCAGTCCAGCAAAGCTGGGATGCATCAT), amplifying a 370 bp band [21]. Thermal cycling conditions were the same as described previously [21]. In short, thermal cycling comprised an initial RT step at 60 °C for 1 min followed by 50 °C for 45 min, then 45 cycles of denaturation at 94 °C for 15 s, annealing at 55 °C for 30 s, and extension at 68 °C for 30 s, and a final extension at 68 °C for 7 min. After the initial screening step, the full length of the S gene for positive samples was amplified using the SuperScript III One-Step RT-PCR System with Platinum (Life Technologies, Karlsruhe, Germany) with gene-specific primers designed for TEHV, SFSVs, and KARVs (Supplementary Table S1). The temperature profile was the same as pan-phlebovirus screening RT-PCR, except the primer annealing temperature was adjusted to 59 °C. PCR products were sequenced through Sanger sequencing.

### 2.5. Nucleocapsid and Non-Structural Protein Analysis and Phylogenetic Tree Construction

In addition to the sequences obtained in this study, a dataset including SBVs from diverse geographical locations and time points was obtained from GenBank. The full-length of S gene sequences was translated to nucleocapsid (N) and non-structural protein (NS) amino acids (AAs). Then, AA sequences of N and NS proteins were aligned using the CLUSTAL W algorithm, and a phylogenetic tree was constructed using the neighbour-joining (NJ) method and p-distance model with sorted topologies and a 70% threshold using the MEGA 11 program with 1000 bootstrap pseudoreplications.

## 3. Results

### 3.1. Prevalence of SBVs in Sandfly Specimens in Iran

A total of 29,216 sandfly specimens were collected (blood-fed and non-blood-fed) at seven trapping sites in Iran during 2019 and 2020. Individual specimens were pooled into 2920 pools and subsequently into 292 super pools. Four super pools were revealed as SBV-positive during pan-phlebovirus RT-PCR. In order to determine which sandfly species were positive, each positive super pool was opened into individual samples. We found that one *Ph. sergenti* collected from Lorestan in August 2019 was positive for TEHV (GenBank accession number: OQ304468), one *Ph. papatasi* collected from East Azerbaijan in June 2020 was positive for an SFSV (OQ304469), and three specimens were positive for KARVs, including two *Ph. papatasi* collected from Tehran in July 2019 (OP850018) and June 2020 (OP850019) and one *Ph. perfiliewi* collected from Tehran in June 2019 (OP850020). All individual positive samples for KARVs were non-blood-fed sandflies (Table 1).

### 3.2. Evolutionary Tree of SBVs Based on N and NS Amino Acids

The full-length sequences of one TEHV, one SFSV, and three KARV S genes are composed of 1308, 1764, and 1686 nt, respectively, and were obtained using primers described in Supplementary Table S1. The genomic organization of the S genes of the TEHV, SFSV, and the three KARV isolates in this study is consistent with other members of SBVs, having two open reading frames (ORFs), in which the ORF closer to the 3′-end of TEHV has a length of 765 nt, SFSV has a length of 786 nt, and KARVs has a length of 726 nt, which encodes the nucleocapsid protein (N protein) containing 254 AA, 261 AA, and 241 AA, respectively. The second ORF is encoded in ambisense and has a length of 429 nt in TEHV, 741 nt in SFSV, and 789 nt in KARVs, which encodes the non-structural protein (NS protein) containing 142 AA, 246 AA, and 262 AA, respectively.

The phylogenetic analysis based on N and NS AA sequences confirmed the clustering of SBV sequences in four main clades/serocomplexes including SFNVs, SFSVs, KARVs and SALVs. Regardless of either N or NS AA sequences, the viruses detected in sandflies in this study fell into three different serocomplexes, specifically SFNVs, SFSVs, and KARVs.

**Figure 2:**
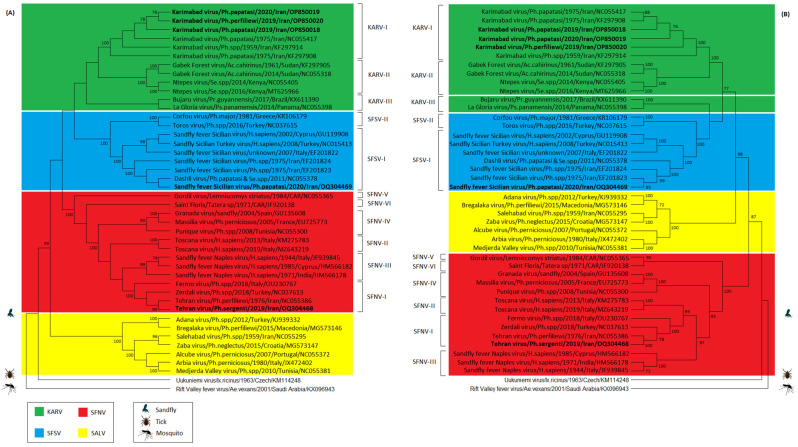
Phylogenetic trees based on the N (**A**) and NS (**B**) protein sequences were constructed with the MEGA software V11. The bootstrap values and number of bootstrap replications were greater than 70% and 1000, respectively. The Uukuniemi virus, a tick-borne phlebovirus, and RVFV, a mosquito-borne phlebovirus, were considered as an outgroup. CAR stands for the Central African Republic. SBV sequences obtained in this study are shown in bold.

A phylogenetic tree based on the N and NS genes showed grouping of SFNV sequences in four distinct lineages. The TEHV sequence obtained from *Ph. sergenti* in the current study fell in lineage I of the SFNV serocomplex and showed highest similarity to a previous TEHV isolated from *Ph. perfiliewi* in 1976 in Iran (NC_055386) and the Zerdali virus from Turkey (KP966618). Both grouped with the Fermo virus from Italy (OU230767), forming lineage I, but remained distinct from other SFNVs in lineages II (Toscana viruses), III (Naples viruses), IV (Granada, Massilia, and Punique viruses), V (Gordil virus), and VI (Saint Floris) (Figure 2).

A phylogenetic tree based on the N and NS genes showed that SFSVs are divided into two main lineages; lineage I includes all SFSVs except the Toros and Corfou viruses, which form lineage II. The SFSV sequence obtained from *Ph. papatasi* in the current study fell within lineage I and was highly similar to previous SFSVs isolated from *Ph.* spp. in 1975 in Iran (EF201823 and EF201824) and the Dashli virus isolated from *Ph. papatasi* and *Se.* spp. in 2011 in Iran (NC_055378). These isolates form sub-lineage Ib, but are distinct from other SFSVs in lineage I originating from Italy, Turkey, and Cyprus, which form sub-lineage Ia (Figure 2).

A phylogenetic tree based on the N and NS genes showed that KARVs are divided into three main lineages based on geographical origins; these include lineage I (isolates from Iran), lineage II (isolates from Africa), and lineage III (isolates from South America). The KARV AA sequences obtained in this study clustered together with KARV sequences previously detected in *Ph. papatasi* in 1975 (NC_055417/KF297911 and KF297908) and *Ph.* spp. in 1959 (KF297914) in Iran. Together, they form lineage I within the KARV serocomplex and are distinct from related strains in lineage II originating from Sudan (Gabek Forest virus; KF297905 and NC_055318) and Kenya (Ntepes virus; MT625966 and NC_055405). The stability of topology between N and NS for KARVs, Gabek Forest virus, and Ntepes virus suggested that these viruses did not contain evidence of recombination. These viruses did, however, group with the Bujaru virus isolated from Brazil (KX611390) and La Gloria virus isolated from Panama (NC_055398) based on the N protein to form lineage III, but not NS protein, indicating a recombination event for the two latter viruses (Figure 2).

## 4. Discussion

SBVs have been documented for over 80 years. Epidemics of SFSVs and SFNVs were first documented among the Allied forces in Italy during World War II [22]. Currently, SFNV and SFSV infections are common in the Mediterranean basin, with seroprevalence rates in some areas nearing 50% [23]. Infections may result in a high temperature, headaches, nausea, vomiting, and diarrhoea [24]. In the Middle East, several SBVs have been identified in phlebotomine sandflies, and many SBVs were originally isolated for the first time in Iran, such as the Iranian SFSV strain from *Ph. papatasi* (probable vector) in 1975, TEHV from *Ph. perfiliewi* in 1976, KARV from *Ph.* spp. in 1959 and *Ph. papatasi* (probable vector) in 1975, and SALV from *Ph.* spp. in 1959 [6,10]. Despite the initial isolation of SFSVs, TEHV, and KARVs from sandfly specimens collected before 1970, subsequent sandfly research in Iran focused on Leishmania, and up until this study, there has been no current published data on SFSV, TEHV, and KARV in Iran. As a result, the role of sandflies as the vector for arboviruses and spread of SBVs in Iran is poorly understood.

Of approximately 800 known sandfly species around the world, Iran has been confirmed to be the habitat of 44 species of sandflies from the two genera *Phlebotomus* and *Sergentomyia* [9]. To estimate the prevalence of different sandfly species in the current study, a parallel study (model host–vector interactions of blood-fed sandflies) that shared the same samples used in this study was conducted, in which a subset of 736 sandflies were identified by targeting the cytb gene for molecular identification [25]. Our results showed that the 736 specimens belonged to ten species, six of which belonged to the genus *Phlebotomus* (n = 465, 63.18%) and four of which belonged to the genus *Sergentomyia* (n = 271, 36.82%). The most common sandfly species was *Ph. papatasi* (n = 283, 38.45%), followed by *Se. sintoni* (n = 267, 36.28%), *Ph. sergenti* (n = 99, 13.45%), *Ph. major* (n = 45, 6.11%), *Ph. alexandri* (n = 14, 1.90%), *Ph. halepensis* (n = 13, 1.77%), *Ph. perfiliewi* (n = 11, 1.49%), *Se. clydei* (n = 2, 0.27%), *Se. iranica* (n = 1, 0.14%)*,* and *Se. tiberiadis* (n = 1, 0.14%) [25]. The data suggest that despite there being 44 different species of sandflies present in Iran, over 70% of the sandflies identified in this sample set belonged to just 2 species: *Ph. papatasi* and *Se. sintoni.* Due to their high prevalence, it is important to understand the potential role (if any) these species have in SBV dissemination to help direct future mitigation plans.

Despite 70% of the sandfly sample set belonging to either *Ph. papatasi* (38.45%) or *Se. sintoni* (36.28%), the proportion of specimens found to carry SBVs did not follow this trend; in fact, no *Sergentomyia* specimens examined in the current study were identified as SBV carriers. Although previous evidence shows the isolation of TEHV from *Ph. perfiliewi*, here, we report *Ph. sergenti* as a potential additional vector for TEHV in Iran. This finding broadens the prior theories that designated *Ph. perfiliewi* as the TEHV vector [3]. With regard to SFSV, we found presence of this SBV in *Ph. papatasi*, a finding that agreed with previous studies in which an SFSV was isolated in Iran from an unidentified *Ph.* spp., with data suggesting that *Ph. papatasi* was the vector. Further studies would need to be undertaken to fully elucidate if the vector of SFSVs is indeed *Ph. papatasi*, as suggested by this study. Our data also seem to confirms the previous assumption that *Ph. papatasi* is associated with KARVs [11,12]. Interestingly, we also report the *Ph. perfiliewi* as another potential vector for KARVs in Iran. Altogether, the previous and current results appear to confirm previously published suggestions that SBVs do not seem to have a very restricted vector association [3]. Although we had a considerable number of species belonging to the genus *Sergentomyia*, no viruses were detected in this genus, nor in other species of the genus *Phlebotomus.* Additional research is needed to further elucidate if there is a role between sandfly species and SBV association, especially as current results suggest this may not be the case.

The identification of SBVs in sandfly vectors in Iran is not surprising as SBVs have previously been identified in the country and are thought to be circulating. A seroprevalence study on samples collected from 1987 to 1988 in western Iran (Ilam and Kermanshah provinces) showed previous exposure to SFSVs and SFNVs in 60% and 46% of the studied population (suspected patients), respectively [26]. Another seroprevalence study on SFVs in western Iran (Ilam province) showed that SFSVs and SFNVs were the most common serotypes among the studied population (201 individuals), with 10.9% and 5% prevalence, respectively [2]. In addition, since the first isolation of KARVs in Iran, serological evidence of human infection has been reported from Iran and some of its neighbouring countries [6,11]. However, it must be noted that due to the potential of cross-reactions between closely related phleboviruses [27], seroprevalence data should not be regarded as proof of the current circulation of SFNVs, SFSVs, or KARVs in Iran, and further studies need to be undertaken to fully elucidate virus circulation in Iran.

On the other hand, our results here based on the molecular detection of SFSVs, TEHV, and KARVs in Iranian provinces are consistent with the earlier molecular and serological studies of these viruses in Iran [2,26], strongly suggesting the circulation of SBVs. Our findings also suggest that earlier assumptions that SFNV is circulating in Iran based on serological data may be erroneous, as it was not detected in this study [3]. Instead, we detected the presence of TEHV, and since TEHV and SFNV share the same antigenic complex and are both members of the SFNV serocomplex, immunochemistry methods such as ELISA or immunofluorescence assays are unable to distinguish between the antibodies induced by these viruses [27]. Furthermore, this could explain why previous reports only discussed the indirect identification of SFNV through antibody detection but not through direct virus isolation [11]. In addition, the phylogenetic tree results from this study based on the N and NS protein sequences also appear to provide evidence that SFNV is not circulating in Iran. The phylogenetic tree based on the N and NS proteins demonstrated that members of SFNVs are classified into six distinct lineages, while the TEHV sequence obtained in the current study formed a distinct cluster with the previous isolate of TEHV (NC_055386), Zerdali virus from Turkey (KP966618), and Fermo virus from Italy (OU230767), separate from other SFNVs in lineages II (Toscana viruses), III (Naples viruses), IV (Granada, Massilia and Punique viruses), V (Gordil virus), and VI (Saint Floris).

The current phylogenetic tree results from this study concur with the previous reports of SFSV and KARV presence in Iran. The SFSV complex comprises SFSVs with diverse origins as lineage I and the Toros and Corfou viruses as lineage II [28]. The SFSV sequence obtained in the current study fell into lineage Ib and grouped with previous isolates of SFSVs (EF201823 and EF201824) and the Dashli virus (NC_055378) from sandflies in Iran. As proposed by Alkan et al. in 2017 [3], SFSV isolates from Iran forming lineage Ib could tentatively be named the Dashli virus. The SBVs within the KARV serocomplex divide into three lineages based on their geographical origins, including lineage I, encompassing KARV with geographical origins in Iran, lineage II, encompassing the Gabek Forest and Ntepes viruses with geographical origins in Africa (e.g., Sudan and Kenya), and lineage III, encompassing the Bujaru and La Gloria viruses from South America (e.g., Brazil and Panama). KARV sequences obtained in the current study formed a monophyletic clade in a sister relationship with other viruses in lineages I and II. Altogether, the molecular data from this study support previous findings of SBV circulation in Iran, although future studies are needed to clarify the impact of SBVs in Iran and determine if mitigation strategies are or will be needed.

Compounding the spread of SBVs in Iran is the known circulation of the virus in countries surrounding Iran [29]. In southern Iraq, a study examining arboviruses detected antibodies in human serum for both SFSVs and SFNVs [30]. In 2007, an outbreak of SFSVs occurred on a military base, affecting Americans in central Iraq [31]. Sandfly fever was also diagnosed in military personnel in Afghanistan in 2017 [32]. The spread of SBVs is a public health concern in several parts of the world, with a more recent example of KARVs being identified in gerbils in China [33]. Therefore, future investigations on SBVs are needed to elucidate the role of SBVs, sandflies, and potential alternate reservoirs of SBVs in countries such as Iran, as many unknowns still exist.

## 5. Conclusions

This was a comprehensive study with a large sample size undertaken to illustrate the current climate of SBVs and their potential vectors in Iran. While most of our findings were in accordance with previous serological evidence of exposure to SBVs in human populations in Iran, it also identified potential pitfalls with using serological data to determine exposure to SBVs, especially with SBVs that fall together within larger serocomplexes. In these situations, molecular data may be more suited to distinguish between viruses of similar origin. The current study provides solid evidence to tackle knowledge gaps in the molecular epidemiology of TEHV, SFSVs, and KARVs in Iran. Our data confirmed the autochthonous transmission and circulation of several SBVs in Iran, including TEHV, SFSVs, and KARVs, and we have provided the first evidence for the circulation of TEHV in *Ph. sergenti* in western Iran. We also determined that *Ph. papatasi* is potentially responsible for the circulation of SFSVs in western Iran, and confirmed that *Ph. papatasi* and *Ph. perfiliewi* are possible vectors carrying KARVs in central Iran. The data from this study provide strong evidence of the circulation of SBVs in Iran, but more studies are needed to fully elucidate the dissemination of SBVs within the country, especially studies targeting full-genome sequencing and virus isolation to further clarify the influence of SBV circulation in Iran.

## Figures and Tables

**Table 1:** Distribution of positive sandfly species for TEHV, SFSV, and KARVs in Iran during the period 2019–2020.

Phlebovirus	Phlebovirus Species	Collection Site	Isolation Year	GenBank Accession #
TEHV	*Ph. sergenti*	Lorestan	August, 2019	OQ304468
SFSV	*Ph. papatasi*	East Azerbaijan	June, 2020	OQ304469
KARV	*Ph. papatasi*	Tehran	July, 2019	OP850018
KARV	*Ph. perfiliewi*	Tehran	June, 2019	OP850020
KARV	*Ph. papatasi*	Tehran	June, 2020	OP850019

Note: Symbol # equals number.

## Data Availability

All relevant data are within the paper and its supporting information file.

## Supplementary Materials

The following supporting information can be downloaded at: https://www.mdpi.com/article/10.3390/microorganisms11112754/s1, Table S1: Primers used for full-length sequencing of S gene of SBVs.

## References

[1] Parhizgari N., Piazak N., Mostafavi E. (2021). Vector-borne diseases in Iran: Epidemiology and key challenges. Future Microbiol..

[2] Shiraly R., Khosravi A., Farahangiz S. (2017). Seroprevalence of sandfly fever virus infection in military personnel on the western border of Iran. J. Infect. Public Health.

[3] Alkan C., Moin Vaziri V., Ayhan N., Badakhshan M., Bichaud L., Rahbarian N., Javadian E.-A., Alten B., de Lamballerie X., Charrel R.N. (2017). Isolation and sequencing of Dashli virus, a novel Sicilian-like virus in sandflies from Iran; genetic and phylogenetic evidence for the creation of one novel species within the *Phlebovirus* genus in the *Phenuiviridae* family. PLoS Negl. Trop. Dis..

[4] Alkan C., Bichaud L., De Lamballerie X., Alten B., Gould E.A., Charrel R.N. (2013). Sandfly-borne phleboviruses of Eurasia and Africa: Epidemiology, genetic diversity, geographic range, control measures. Antivir. Res..

[5] Mazelier M., Rouxel R.N., Zumstein M., Mancini R., Bell-Sakyi L., Lozach P.-Y. (2016). Uukuniemi virus as a tick-borne virus model. J. Virol..

[6] Palacios G., Tesh R.B., Savji N., da Rosa A.P.T., Guzman H., Bussetti A.V., Desai A., Ladner J., Sanchez-Seco M., Lipkin W.I. (2014). Characterization of the Sandfly fever Naples species complex and description of a new Karimabad species complex (genus *Phlebovirus*, family *Bunyaviridae*). J. Gen. Virol..

[7] Marklewitz M., Tchouassi D.P., Hieke C., Heyde V., Torto B., Sang R., Junglen S. (2020). Insights into the evolutionary origin of Mediterranean sandfly fever viruses. MSphere.

[8] Alkan C., Erisoz Kasap O., Alten B., de Lamballerie X., Charrel R.N. (2016). Sandfly-borne phlebovirus isolations from Turkey: New insight into the *Sandfly fever Sicilian* and *Sandfly fever Naples* species. PLoS Negl. Trop. Dis..

[9] Karimi A., Hanafi-Bojd A.A., Yaghoobi-Ershadi M.R., Akhavan A.A., Ghezelbash Z. (2014). Spatial and temporal distributions of phlebotomine sand flies (Diptera: Psychodidae), vectors of leishmaniasis, in Iran. Acta Trop..

[10] Palacios G., Savji N., Travassos da Rosa A., Desai A., Sanchez-Seco M.P., Guzman H., Lipkin W.I., Tesh R. (2013). Characterization of the Salehabad virus species complex of the genus *Phlebovirus* (*Bunyaviridae*). J. Gen. Virol..

[11] Tesh R., Saidi S., Gajdamovič S.J., Rodhain F., Vesenjak-Hirjan J. (1976). Serological studies of the epidemiology of sandfly fever in the Old World. Bull. World Health Organ..

[12] Tesh R., Saidi S., Javadian E., Nadim A. (1977). Studies on the epidemiology of sandfly fever in Iran. I. Virus isolates obtained from *Phlebotomus*. Am. J. Trop. Med. Hyg..

[13] Saidi S., Tesh R., Javadian E., Sahabi Z., Nadim A. (1977). Studies on the epidemiology of sandfly fever in Iran. II. The prevalence of human and animal infection with five phlebotomus fever virus serotypes in Isfahan province. Am. J. Trop. Med. Hyg..

[14] Darwish M.A., Hoogstraal H., Roberts T.J., Ghazi R., Amer T. (1983). A sero-epidemiological survey for Bunyaviridae and certain other arboviruses in Pakistan. Trans. R. Soc. Trop. Med. Hyg..

[15] Gaidamovich S., Baten M., Klisenko G., Melnikova Y. (1984). Serological studies on sandfly fevers in the Republic of Bangladesh. Acta Virol..

[16] Gaidamovich S.I., Obukhova V.R., Sveshnikova N.A., Cherednichenko I.N., Kostiukov M.A. (1978). Natural foci of viruses borne by *Phlebotomus papatasi* in the USSR according to a serologic study of the population. Vopr. Virusol..

[17] Shahhosseini N., Moosa-Kazemi S.H., Sedaghat M.M., Wong G., Chinikar S., Hajivand Z., Mokhayeri H., Nowotny N., Kayedi M.H. (2020). Autochthonous transmission of West Nile virus by a new vector in Iran, vector-host interaction modeling and virulence gene determinants. Viruses.

[18] Theodor O., Mesghali A. (1964). On the phlebotominae of Iran. J. Med. Entomol..

[19] Shahhosseini N., Kayedi M.H., Sedaghat M.M., Racine T., Kobinger G.P., Moosa-Kazemi S.H. (2018). DNA barcodes corroborating identification of mosquito species and multiplex real-time PCR differentiating *Culex pipiens* complex and *Culex torrentium* in Iran. PLoS ONE.

[20] Absavaran A., Rassi Y., Parvizi P., Oshaghi M., Abaie M., Rafizadeh S., Mohebali M., Zarea Z., Javadian E. (2009). Identification of sand flies of the subgenus *Larroussius* based on molecular and morphological characters in North Western Iran. J. Arthropod. Borne Dis..

[21] Lambert A.J., Lanciotti R.S. (2009). Consensus amplification and novel multiplex sequencing method for S segment species identification of 47 viruses of the Orthobunyavirus, Phlebovirus, and Nairovirus genera of the family Bunyaviridae. J. Clin. Microbiol..

[22] Lvov D.K., Shchelkanov M.Y., Alkhovsky S.V., Deryabin P.G., Lvov D.K., Shchelkanov M.Y., Alkhovsky S.V., Deryabin P.G. (2015). Chapter 8—Single-Stranded RNA Viruses. Zoonotic Viruses in Northern Eurasia.

[23] Dionisio D., Esperti F., Vivarelli A., Valassina M. (2003). Epidemiological, clinical and laboratory aspects of sandfly fever. Curr. Opin. Infect. Dis..

[24] Hornak K.E., Lanchy J.-M., Lodmell J.S. (2016). RNA Encapsidation and Packaging in the Phleboviruses. Viruses.

[25] Shahhosseini. N., Sedaghat M.M. (2023). Shahhosseini. N.; Sedaghat, M.M.; Paquette, S-J.; Abai, M.R; Kayedi, M.H. Genotyping, bionomics and host-feeding behavior of *Phlebotomus* spp. (Diptera: Psychodidae) in Iran. Zool. Anzeiger..

[26] Tavana A.M. (2001). The seroepidemiological studies of sand fly fever in Iran during imposed war. Iran. J. Public Health.

[27] Bichaud L., Izri A., De Lamballerie X., Moureau G., Charrel R. (2014). First detection of Toscana virus in Corsica, France. Clin. Microbiol. Infect..

[28] Palacios G., Savji N., Travassos da Rosa A., Guzman H., Yu X., Desai A., Rosen G.E., Hutchison S., Lipkin W.I., Tesh R. (2013). Characterization of the Uukuniemi virus group (*Phlebovirus*: *Bunyaviridae*): Evidence for seven distinct species. J. Virol..

[29] Mostafavi E., Ghasemian A., Abdinasir A., Nematollahi Mahani S.A., Rawaf S., Salehi Vaziri M., Gouya M.M., Nguyen T.M.N., Al Awaidy S., Al Ariqi L. (2021). Emerging and Re-Emerging Infectious Diseases in the WHO Eastern Mediterranean Region, 2001–2018. Int. J. Health Policy Manag..

[30] Barakat A.M., Smura T., Kuivanen S., Huhtamo E., Kurkela S., Putkuri N., Hasony H.J., Al-Hello H., Vapalahti O. (2016). The Presence and Seroprevalence of Arthropod-Borne Viruses in Nasiriyah Governorate, Southern Iraq: A Cross-Sectional Study. Am. J. Trop. Med. Hyg..

[31] Ellis S.B., Appenzeller G., Lee H., Mullen K., Swenness R., Pimentel G., Mohareb E., Warner C. (2008). Outbreak of SandFly Fever in Central Iraq, September 2007. Mil. Med..

[32] Downs J.W., Flood D.T., Orr N.H., Constantineau J.A., Caviness J.W. (2017). Sandfly fever in Afghanistan-a sometimes overlooked disease of military importance: A case series and review of the literature. US Army Med. Dep. J..

[33] Li Y., Wang Y.-N., Tian F., Zhang X.-L., Zhang J.-T., Li S., Li H., Zhang X.A., Liu W. (2022). First report of Karimabad virus in *Rhombomys opimus* in China. One Health.

