# Supplementary material for: Genetic Characterization of Sandfly-Borne Viruses in Phlebotomine Sandflies in Iran
Source: Microorganisms. 2023 Nov 11;11(11):2754. doi: 10.3390/microorganisms11112754 (PMC10673459; doi:10.3390/microorganisms11112754)
Supplement: Supplementary file 1 [file microorganisms-11-02754-s001.zip › microorganisms-2660113-supplementary.pdf]

Supplementary Table S1. Primers used for full-length sequencing of S gene of SBVs.

	Primers	Sequence (5'->3')	Product size (bp)
KARV	KARV-F1	ACACAAAGCTTCCCTGAAATACA	779
	KARV-R1	GCGGCTTTAAGCAAGAGAGC	Tm 58.5-60
	KARV-F2	ATTGACATGAACCTGAAGCCC	519
	KARV-R2	GAGCCCAAGTCTGGAGGAGT	Tm 58.5-60.1
	KARV-F3	CATCGTGATCTTCTTATGGAGGGA	549
	KARV-R3	CCTCCCTGCTTGATCACAATTC	Tm 59.7-59.3
TEHV	TEHV-F1	AAGATTCCCGTTTACTAACTGACT	566
	THEV-R1	AAGAGTCCATGGGCATCAGC	Tm 57-60
	TEHV-F2	AAGAGCCATGATGCATCCGA	858
	THEV-R2	CCAAAGCCAACAATTCAATAGTCAT	Tm 58-59
SFSV	SFSV-F1	CCCAAACGATGAATAGCCAGT	737
	SFSV-R1	CACCCATCGGGAGTTTCCAT	Tm 58.3-59.5
	SFSV-F2	CCTATCGGATGGATGACCCT	561
	SFSV-R2	GCAATTCTGGATGCCCACAAA	Tm 58-59.7
	SFSV-F3	CAATGTCTCTCTTGGAGGCCC	621
	SFSV-R3	ACACAAAGGTCCCTAGTTAATCTG	Tm 58-60.4
